# Executive Functioning and Language in a Pediatric Population with Autism Spectrum Disorders and Epilepsy: A Comparative Study

**DOI:** 10.3390/children11030306

**Published:** 2024-03-05

**Authors:** Alejandro Cano-Villagrasa, Nadia Porcar-Gozalbo, Isabel López-Chicheri, Miguel López-Zamora

**Affiliations:** 1 Facultad de Ciencias de la Salud, Universidad Internacional de Valencia, 46002 Valencia, Spain; nadia.porcar@professor.universidadviu.com; 2 UCAM Universidad Católica de Murcia, 30107 Murcia, Spain; ilchicheri@ucam.edu; 3 Departamento de Psicología Evolutiva y de la Educación, Universidad de Málaga, 29010 Málaga, Spain; miglopzam@uma.es

**Keywords:** autism, childhood, language, executive functions, epilepsy

## Abstract

In recent years, there has been an increase in the prevalence of comorbidity between ASD and epilepsy in the pediatric population. Children with ASD and epilepsy often exhibit greater impairments in executive functions such as cognitive flexibility, planning, inhibition, and emotional control, as well as in language dimensions such as phonology, semantics, morphosyntax, and pragmatics. These impairments can significantly impact their maturation and development. The aim of this study was to assess and compare the executive functioning and language skills of 150 participants, divided into three groups: one with ASD only, another with epilepsy only, and the third group with both ASD and epilepsy. The study utilized the Behavior Rating Inventory of Executive Function (BRIEF-2) and Neuropsychological Evaluation of Executive Functions in Children (ENFEN) to assess executive functions, and Clinical Evaluation of Language Fundamentals 5 (CELF-5) to evaluate language skills. The results indicated that participants with this comorbidity had lower scores in both executive functioning and language skills compared to children with only ASD or epilepsy. The presence of epilepsy significantly limits the executive and linguistic performance of children with ASD, negatively affecting language acquisition, functionality, and the ability to carry out basic life activities independently.

## 1. Introduction

The study of the comorbidity between Autism Spectrum Disorder (ASD) and epilepsy is a rapidly growing field of research due to its increasing prevalence and the limitations imposed on the affected pediatric population, which go beyond those caused by the two disorders separately [1]. ASD is characterized by limitations in social interaction and repeated and stereotyped behaviors [2]. It presents a variety of symptoms and developmental alterations, including deficiencies in cognitive and language dimensions. Difficulties in expressive language, such as pragmatics and understanding writing are found [3]. Intelligence, executive functions, memory, and attention may also be affected [4].

Research on epilepsy in ASD is not new. As early as 1943, Leo Kanner observed that the presence of epileptic seizures in children with autism could negatively impact the acquisition of their language and social skills [5]. Currently, routine screening protocols have been established by epilepsy specialist neurologists to detect this comorbidity early, as early detection is a good prognostic factor for the development of cognitive-linguistic and social skills in children with epilepsy and ASD, especially if both disorders manifest in the early preschool years [6]. Additionally, families often participate in the evaluation process, as they can detect the possible occurrence of epileptic episodes [7,8].

ASD has a significant prevalence, with data from the Centers for Disease Control and Prevention (CDC) in the United States estimating that approximately 1 in 44 children are affected by this condition [8,9]. Globally, the World Health Organization (WHO) reports an average prevalence of around 1% for ASD [7,8,9,10]. On the other hand, epilepsy, a disease with prevalence variations depending on the region and age, affects approximately 50 million people worldwide, with rates ranging from 0.5% to 1% of the general population, according to the WHO [9,10,11,12]. The coexistence of ASD and epilepsy presents specific rates that can vary depending on the studied population and the methodology used in the research, reporting that between 5% and 38% of individuals with ASD also experience epilepsy, according to various studies [8,9,10,11].

### 1.1. Cognitive-Linguistic Profile of Epilepsy

In terms of executive functions, epilepsy influences various neural components. Inhibition, crucial for impulse control and concentration on specific tasks, may experience disruptions, manifesting in challenges to restrain inappropriate behaviors or disruptive conduct [12,13,14]. Cognitive flexibility, one of the fundamental factors for adapting to changes is presented as a potentially vulnerable area, affecting the ability to transition smoothly between different tasks or situations [13,14,15]. Emotional control, essential for emotional regulation, may be affected, contributing to variations in mood [12]. In terms of planning, the ability to organize sequences of actions and establish goals may experience difficulties [13]. The organization and supervision of material, crucial elements in the management of daily tasks, also present alterations, with implications for autonomy and efficiency in everyday activities [16,17].

An analysis of the linguistic profile of these populations reveals the existence of linguistic difficulties associated with pediatric epilepsy, encompassing phonology, semantics, morphosyntax, pragmatics, and oral comprehension [13]. In the phonological domain, there is the possibility of alterations in speech sound production, leading to complications in articulation and auditory discrimination, phenomena directly linked to the variability in the frequency and severity of seizures [11,12,13]. Semantically, the acquisition and organization of word meanings seem disrupted, directly affecting the understanding of abstract concepts [10]. Morphosyntactic difficulties indicate variations in grammatical expression, while in the pragmatic domain, crucial for social communication, children affected by epilepsy may manifest obstacles in using language appropriately in social contexts, significantly affecting the quality of interactions [14]. Regarding comprehension, the interpretation of verbal instructions and text comprehension may be compromised, generating additional challenges in both academic and social environments [15].

### 1.2. Cognitive-Linguistic Profile of ASD

Regarding executive functions, ASD implies additional challenges. Problems in inhibition, necessary for impulse control and maintaining focus on specific tasks, have been found, resulting in difficulties in stopping unwanted behaviors [11,12,13,14]. Cognitive flexibility may also be altered, affecting the ability to transition smoothly from one task to another [8,9,10,11]. The presence of difficulties in emotional control has also been studied, contributing to mood variability and emotional expression [14,15,16]. Lastly, in terms of planning, the ability to organize sequences of actions and establish goals may be compromised [15,16,17,18], affecting the organization and supervision of materials, the pillars of task management [17].

The linguistic profile of individuals with ASD can be complex and varied, with similarities and differences compared to epilepsy. Although both disorders share certain aspects, ASD presents specific symptoms that require detailed analysis. For example, in the phonological domain, they face challenges such as articulatory difficulties and auditory discrimination, which can affect both comprehension and verbal expression [10]. In the semantic domain, alterations in the acquisition and organization of word meanings are observed, influencing the understanding of abstract concepts [13,14,15,16,17,18]. Additionally, at the morphosyntactic level, they may exhibit variations in grammatical expression. These difficulties are reflected in the pragmatics of language, where individuals with ASD may struggle to use language appropriately in social contexts, affecting their social interactions and the comprehension of verbal instructions and texts [12,13,14,15,16,17]. Although some characteristics may be shared with other disorders such as epilepsy, ASD is distinguished by its unevenness in the linguistic profile, with more pronounced weaknesses in pragmatics and strengths in phonological development. Understanding these differences is essential to provide appropriate interventions and support for individuals with ASD, recognizing that not all experience the same difficulties.

### 1.3. Cognitive-Linguistic Profile of ASD with Epilepsy

As for deficiencies in executive functions in participants with ASD and epilepsy, 50% experience delays in learning due to various factors such as syndrome type, etiology, age of onset, frequency and duration of seizures, family history, psychiatric comorbidity, and treatment-related factors [18,19,20,21]. These alterations are directly related to basic executive functions such as cognitive flexibility, planning, emotional control, monitoring, flexibility, working memory, and material organization [22,23,24]. On one hand, children with ASD and epilepsy may face difficulties with cognitive flexibility, planning, and organization when initiating or completing tasks. They may also struggle to organize their belongings and study materials, leading to frustration due to the inability to perform these activities independently [25,26].

On the other hand, deficits in working memory may be observed when completing activities, following instructions, and remembering recent information, important details, and information necessary to correctly complete an activity or task [27]. Research such as that by Papazian et al. [28] and Diamond [29] indicates that children with ASD and epilepsy experience difficulties in inhibition, manifested in impulsive reactions without thought, initiating activities without considering factors to achieve goals or speaking without waiting for their turn. Additionally, emotional control and regulation have been identified as one of the main areas affected in individuals with comorbid ASD and epilepsy. This population may experience difficulties in self-regulation, impulse management, problem recognition, and interpretation of emotions [30,31]. Difficulties in emotional regulation can lead to increased stress in response to new situations, sensory stimuli, or mood changes, resulting in irritability, lack of anger control, tantrums, and self-injurious behaviors [32]. Therefore, establishing daily routines with a structured environment for each activity is crucial to support individuals with this comorbidity [33].

An area significantly affected by the presence of comorbidity between ASD and epilepsy is language. However, research on linguistic competence in this population is limited. A study by Kuczynski [34] on children with ASD and focal epilepsy found a high incidence of language impairment, especially in the areas of semantics, morphosyntax, and pragmatics. Similarly, a study by Requero and Bermejo [35] showed that early-onset focal epilepsy in childhood has a negative impact on the development of expressive and receptive language due to cortical structural alterations in ventral and temporal pathways. This indicates that children with this comorbidity may have poor performance in language skills [36]. Ruggieri [37] attributes this to a higher prevalence of left hemisphere involvement in children with right dominance and early-onset epileptic episodes.

### 1.4. Comparison of Executive Functioning and Language in the Population with Epilepsy, ASD, and ASD with Epilepsy

A detailed comparison of executive functioning and language skills among populations with epilepsy, ASD, and ASD with epilepsy reveals distinctive nuances that underscore the complexities associated with each group. In the case of epilepsy, challenges in executive functioning are observed, especially after seizure episodes, with notable impacts on working memory and selective attention [33,34,35,36]. Linguistically, variability in the frequency and severity of seizures can influence difficulties in language comprehension and expression. On the other hand, ASD presents a unique profile with specific challenges in inhibition, cognitive flexibility, and emotional control, affecting adaptability and emotional regulation [38].

Language difficulties in ASD encompass challenges in social communication, organization, and expression of meaning, with notable pragmatic alterations and a tendency towards literal language interpretation. When examining the population with ASD and epilepsy, the intersection of both conditions generates a combination of challenges in both executive functioning and language skills [36,37,38]. Executive difficulties, such as inhibition, cognitive flexibility, and emotional control, may experience intensification, affecting adaptability and emotional regulation more profoundly. Linguistically, the coexistence of ASD and epilepsy could lead to a combination of challenges in social communication and language processing capacity, exacerbating pragmatic alterations and literal language interpretations [39].

### 1.5. Justification of the Research

This research arises from the need to explore and compare the inherent complexities in explaining difficulties in executive and linguistic functioning in the population with ASD and epilepsy. As described earlier, current scientific literature indicates that the comorbid condition of ASD and epilepsy complicates the linguistic and executive functioning profiles of children with these two conditions, significantly limiting maturation, learning, and integration into the environments and contexts where individuals with ASD and epilepsy live. However, it is important to note that there is a limited number of published studies about linguistic and executive functioning in this population. Additionally, there are no articles comparing language and executive functioning among these three clinical profiles, except for the recently published article by Cano-Villagrasa et al. [40], which only measures executive functioning and compares individuals with epilepsy, ASD, or ASD and epilepsy. Findings suggest that participants diagnosed with epilepsy demonstrate better performance in tasks related to executive functions compared to individuals with ASD and ASD with epilepsy.

However, other comparative studies attempt to describe the profiles of linguistic and executive functioning in these populations without directly comparing them. For example, studies conducted by Barboza Ubarnes et al. [41], Pisón et al. [25], del Busto [42], Campos-Castelló and Campos-Soler [36], Zambarbieri [43], Dioses Chocano et al. [44], Juillerat et al. [45], Muñoz-Yunta et al. [6], Norrelgen et al. [46], and Rotta [47] provide an exposition of expressive and receptive language characteristics, as well as cognitive and executive competencies in individuals with epilepsy, ASD, or ASD with epilepsy, describing existing alterations in these populations. From these studies, it can be inferred that the profiles of children with epilepsy will show better performance in tasks related to language and executive functioning compared to children diagnosed with ASD or ASD comorbid with epilepsy. Therefore, this research emphasizes that, due to the absence of recent investigations, the importance of our work is increased, as it provides a valuable contribution to understanding the difficulties in executive and linguistic functioning in this specific group of children.

## 2. Objectives and Research Hypothesis

Consequently, the main objective of this study is to assess and compare deviations in executive functioning and language within three distinct groups: children diagnosed with ASD, children diagnosed with epilepsy, and children diagnosed with both ASD and epilepsy. Currently, there is a scarcity of research systematically contrasting linguistic competence and executive functioning in children facing this comorbidity. Within this framework, regarding the comparison between these three groups, the general hypothesis posited that children with epilepsy would demonstrate better cognitive and linguistic performance than groups composed of children diagnosed with ASD or ASD and epilepsy. Thus, the first hypothesis proposed for this study is that executive functioning skills will be superior in the group of children with epilepsy compared to the group of individuals with ASD, followed by the group of ASD with epilepsy [20,40,41,43,44,45,46]. Similarly, the second research hypothesis posited that performance in language dimensions is higher in the group of children with epilepsy compared to the group of children with ASD and the group of children with ASD with epilepsy [14,17,18,22,47,48,49,50].

## 3. Materials and Methods

### 3.1. Participants

The study involved 150 children aged 6 to 8 years, categorized into three groups: ASD (*n* = 50), epilepsy (*n* = 50), and ASD with epilepsy (*n* = 50). Within the ASD group (G1), there were 24 boys and 26 girls (Mean Age = 6.8; SD = 0.9) diagnosed with ASD levels 2 and 3 according to DSM-5 criteria (63% and 37%, respectively). The epilepsy group (G2) comprised 28 boys and 22 girls (Mean Age = 7.4; SD = 0.6). Lastly, the ASD with epilepsy group (G3) included 31 boys and 19 girls (Mean Age = 7.2; SD = 0.8) diagnosed with ASD level 3 based on DSM-5 criteria. This age range was selected because the assessment tools used in the study are suitable for children aged 6 and above, and it represents a stage where diagnoses are typically established. All participants in the G2 and G3 groups received a diagnosis of generalized epilepsy. To ensure homogeneity in the number of participants between groups, each group was configured with a total of 50 participants. This process involved refining the database that recorded the participants’ scores and standardizing the number of subjects to 50 per group.

All participants in this study were diagnosed with ASD or epilepsy in the Mental Health Units or Neuropediatrics services of reference hospitals in their respective cities. The diagnosis of ASD and/or epilepsy was conducted by an interdisciplinary team composed of a psychiatrist, a pediatrician, a neurologist, and a psychologist at the hospital in their locality. On one hand, the diagnosis of ASD was made using the M-CHAT-R instrument [51]. Children with scores indicating suspected ASD underwent additional assessment using the ADOS-2. Autism Diagnostic Observation Schedule—2 [52] and ADI-R. Autism Diagnostic Interview—Revised [53] protocols to confirm ASD diagnosis. The diagnostic criteria established in DSM-5 for ASD and the level of needs presented by the participants in this study were also followed. Furthermore, individuals displaying symptoms of epilepsy underwent neurological assessments, including nuclear magnetic resonance, private sleep electroencephalogram, and genetic testing to confirm the diagnosis. The participants in this study exhibited a level of disability characterized by limitations or obstacles hindering their full participation in society in a functional capacity. Lastly, all participants underwent evaluation using the Wechsler Intelligence Scale for Children-IV (WISC-IV) [54], with exclusion criteria based on an IQ below 60 (Table 1).

Specific inclusion and exclusion criteria were defined. Inclusions covered individuals within the age range of 6 to 8 years, along with a confirmed diagnosis of ASD or epilepsy. Conversely, exclusions encompassed individuals meeting any of the following conditions: (I) presenting a motor or sensory ailment that hindered or obstructed the precise execution of the study; (II) lacking any form of communication or language competence before the age of 5 and (III) all those participants who scored below 60 on the WISC-IV assessment test, indicating a level of intellectual disability categorized as moderate, severe, or profound. Finally, sample selection was carried out by identifying participants with a diagnosis of ASD, epilepsy, or ASD with epilepsy. A total of 232 participants were obtained, of which 82 were excluded for not meeting the inclusion and exclusion criteria. Therefore, a sample of 150 participants was configured and divided into three groups of 50 subjects each, forming the study’s sample group.

### 3.2. Instruments and Materials

#### 3.2.1. Behavior Rating Inventory of Executive Function

Behavior Rating Inventory of Executive Function (BRIEF-2) [55] is an instrument that provides an assessment of executive functions in participants aged 5 to 18. It measures different aspects of executive functioning more precisely than other available tests and instruments. BRIEF-2 responds to the highlighted need for an ecologically valid method to evaluate the molar, behavioral, and everyday dimensions and aspects of executive functions in participants. It is completed in an estimated time of 10 min. There are two versions of the questionnaire, one completed by the primary caregivers and the other by the tutor at the educational center the child attends. The Cronbach’s alpha value is 0.82. In this study, the dependent variables included for data analysis were: Inhibition, Flexibility, Emotional Control, Working Memory, Planning, Organization of Materials, and Monitoring.

#### 3.2.2. ENFEN—Neuropsychological Evaluation of Executive Functions in Children

The ENFEN Neuropsychological Evaluation of Executive Functions in Children tool, translated as Neuropsychological Evaluation of Executive Functions in Children [56], assesses cognitive performance in activities related to executive functions in children and their level of maturity through four tests (interference resistance, path construction, verbal fluency, and ring construction). Regarding its psychometric properties, Rodríguez et al. [57] conducted a measurement of these factors. The Cronbach’s alpha value is 0.81. In this study, the dependent variables included for data analysis were: Verbal Fluency, Path Construction, Ring Construction, and Interference Resistance.

#### 3.2.3. Clinical Evaluation of Language Fundamentals 5

Clinical Evaluation of Language Fundamentals 5 (CELF-5) [58] is an individual clinical assessment tool designed to identify, diagnose, and monitor language and communication disorders in children and adolescents aged 5 to 15 years. This instrument aims to evaluate the strengths and weaknesses of the participant in different aspects and dimensions of language. It is an assessment test that demonstrates high reliability and effectiveness in evaluating the linguistic profile of child and adolescent populations. All these scales are scored on a scale from 0 to 1, where 0 represents the absence of the skill and 1 represents the presence of the skill, except for the linguistic profile questionnaire, which consists of a structured response on a Likert scale from 0 to 4, where 0 is related to never and 4 to always. The Cronbach’s alpha value is 0.89. In this study, the dependent variables included for data analysis were only those listed below, using the raw direct scores of each: Sentence Comprehension, Linguistic Concepts, Word Structure, Semantic Relationships, Following Directions, Formulated Sentences, Recalling Sentences, Understanding Spoken Paragraphs, Word Definitions, Sentence Assembly, and Pragmatics Profile.

### 3.3. Procedure

This research was approved by the ethics committee of the Universidad Católica San Antonio de Murcia (UCAM) with the code CE052206 on 27 May 2022. The study protocol involved an initial evaluation of participants according to the previously established inclusion and exclusion criteria. Subsequently, the sample was divided into three groups: ASD without epilepsy, Epilepsy, and ASD with epilepsy. Participant data were recorded in two assessment sessions, each lasting approximately 60 min. In the first session, participants’ main data were collected through an initial interview, and the ENFEN test was administered to the children, while the BRIEF-2 test was given to the parents. In the second session, the CELF-5 test was administered to the participants. The assessment was conducted by two specialized professionals: a speech and language therapist for language evaluation and a neuropsychologist for executive functioning assessment. All data were recorded in a protected database, with the aim of organizing the data and conducting relevant statistical analyses. Finally, a report with the participants’ assessment results was sent to the parents.

### 3.4. Data Analysis

The analyses in this quasi-experimental cross-sectional study were conducted using the statistical package SPSS (version 25). Firstly, the normality of the distributions of the continuous dependent variables was examined using the Chi-Square test to observe the differences in each of the sociodemographic variables characterizing the sample group. After analyzing the homogeneity of variances, homoscedasticity was found among the sociodemographic variables of the participants. Secondly, multivariate analysis of covariance (MANCOVA) analyses were performed to compare language skills and variables related to executive functioning among the groups to observe differences using the intellectual disability variable as a covariate, due to significant differences in the distribution of this variable in the sample of the present study (*n* = 27; participants with intellectual disability scoring between 60 and 70 on the IQ scale). The IQ is distributed as follows in the different study groups; ASD group [M = 89.2; SD = 6.23]; Epilepsy group [M = 91.4; SD = 3.54] and ASD with Epilepsy [M = 78.1; SD = 13.57; G3 < G1, G2). To control the probability of a Type I error, a correction factor for adjusted critical *p*-values was introduced when conducting multiple comparisons, employing a reduction method: the Holm–Bonferroni correction [59].

## 4. Results

### 4.1. Difference in Executive Functioning between the Tested Groups

The MANCOVA was conducted to examine variations in executive functioning skills across the ASD, Epilepsy, and combined ASD and epilepsy groups. The analysis revealed significant differences, supported by statistically significant results (Wilks’ Lambda = 0.023, F(14) = 113.195, *p* < 0.001, η^2^_P_ = 0.849). As depicted in Table 2, the variables showing noteworthy distinctions included inhibition, flexibility, emotional control, working memory, planning, organization of materials, and monitoring. The detailed outcomes of the MANCOVAs regarding executive functioning are outlined in Table 2.

The results from Table 2 indicate significant differences in executive functioning measures among the three groups: ASD (G1), Epilepsy (G2), and ASD with epilepsy (G3), as obtained in the BRIEF-2 test. For the Inhibition domain, G3 (M = 4.50, SD = 2.97) showed lower scores compared to G1 (M = 14.86, SD = 3.60) and G2 (M = 23.58, SD = 3.74), with a statistically significant difference (F = 382.16, *p* < 0.05, η^2^_P_ = 0.839), indicating that G3 exhibited the least inhibitory control. Similar patterns were observed in the Flexibility, Emotional Control, Work Memory, Planning, Organization of Materials, and Monitoring domains, with G3 consistently showing lower scores than G1 and G2. These differences were statistically significant for all domains (*p* < 0.05), with large effect sizes (η^2^_P_ ranging from 0.825 to 0.866). The general trend suggests that the executive functioning of children with both ASD and epilepsy (G3) is poorer compared to those with ASD alone (G1) and epilepsy alone (G2), highlighting the potential additive impact of the coexistence of these conditions on various aspects of executive functioning.

Post-hoc analyses revealed significant differences among the ASD, epilepsy, and ASD with epilepsy groups in the variables assessed by the BRIEF-2 test (*p* < 0.001). Across all variables, the epilepsy group showed higher scores in executive functioning subscales compared to the ASD group, followed by the ASD and epilepsy group, which exhibited the most pronounced alterations.

Similarly, a MANCOVA was performed to examine variations in executive functioning skills, as measured by the ENFEN instrument, among the ASD, epilepsy, and combined ASD and epilepsy groups. The analysis revealed significant differences (Wilks’ Lambda = 0.083, F(8) = 87.304, *p* < 0.001, η^2^_P_ = 0.708). As delineated in Table 3, notable differences were observed in variables such as Verbal Fluency, Trail Construction, Ring Construction, and Interference Resistance. Detailed results of the MANCOVAs concerning executive functioning are provided in Table 3.

The results of Table 3 indicate significant differences in executive functioning measures among the three groups: ASD (G1), Epilepsy (G2), and ASD with epilepsy (G3). In verbal fluency, G3 (M = 9.40, SD = 3.37) showed lower scores compared to G1 (M = 16.82, SD = 3.37) and G2 (M = 23.84, SD = 5.25), with a statistically significant difference (F = 155.27, *p* < 0.05, η^2^_P_ = 0.679), indicating that G3 exhibited less fluent verbal performance. In trail and ring constructions, G3 also demonstrated lower scores than G1 and G2, with significant differences in all three cases (F = 191.16, F = 204.40, *p* < 0.05, η^2^_P_ = 0.722, 0.736, respectively), indicating difficulties in visuospatial planning and fine motor skills in G3. In interference resistance, G3 (M = 8.20, SD = 2.99) showed inferior performance compared to G1 (M = 18.22, SD = 3.77) and G2 (M = 24.12, SD = 5.39), with a statistically significant difference (F = 185.84, *p* < 0.05, η^2^_P_ = 0.717), indicating challenges in suppressing interference and maintaining selective attention in G3. These findings support the notion that the coexistence of Autism Spectrum Disorder and epilepsy (G3) is associated with specific deficiencies in various facets of executive functioning compared to the individual groups G1 and G2.

Post-hoc tests showed statistically significant differences between ASD, epilepsy, and ASD with epilepsy groups in the variables comprising the ENFEN test (*p* < 0.001). In all variables, the scores in the subscales measuring executive functioning of the epilepsy group were higher than those obtained in the ASD group, followed by the ASD and epilepsy group. The latter was the one in which greater alterations were observed.

### 4.2. Language Differences between the Tested Groups

The MANCOVA analysis conducted to examine variations in language proficiency measures among ASD, epilepsy, and ASD with epilepsy groups revealed notable distinctions (Wilks’ Lambda = 0.020, F(18) = 94.530, *p* < 0.001, η^2^_P_ = 0.860). As depicted in Table 4, significant differences were observed in Sentence Comprehension, Linguistic Concepts, Word Structure, Semantic Relationships, Following Directions, Formulated Sentences, Recalling Sentences, Understanding Spoken Paragraphs, Word Definitions, Sentence Assembly, and Pragmatics Profile. The outcomes of the MANCOVAs regarding linguistic abilities are outlined in Table 4 below.

The analysis reveals significant differences among the ASD (G1), Epilepsy (G2), and ASD with epilepsy (G3) groups. In terms of Sentence Comprehension, the G3 group exhibits notably lower mean scores (M = 4.44, SD = 2.908) compared to both G1 (M = 14.92, SD = 3.680) and G2 (M = 25.06, SD = 4.133), indicating pronounced challenges in comprehending complex linguistic structures within G3 (F = 408.05, *p* < 0.05, η^2^_P_ = 0.847). Similarly, in Linguistic Concepts, G3 (M = 4.40, SD = 3.117) demonstrates inferior performance relative to both G1 (M = 15.84, SD = 3.776) and G2 (M = 24.40, SD = 3.860), with a significant difference (F = 388.55, *p* < 0.05, η^2^_P_ = 0.841), suggesting difficulties in grasping abstract concepts. Word Structure also presents challenges for G3 (M = 4.12, SD = 3.055), which scores lower than both G1 (M = 16.36, SD = 4.355) and G2 (M = 23.50, SD = 3.477) with a significant difference (F = 356.72, *p* < 0.05, η^2^_P_ = 0.829), indicating difficulties in understanding grammatical structure and syntax. Furthermore, G3 exhibits inferior performance in Semantic Relationships (M = 4.72, SD = 2.865) compared to G1 (M = 16.58, SD = 3.866) and G2 (M = 24.18, SD = 4.183), with a significant difference (F = 354.96, *p* < 0.05, η^2^_P_ = 0.828), suggesting additional challenges in word and concept association.

Additionally, G3 demonstrates lower performance in Following Directions compared to both G1 and G2, with a significant difference (F = 419.14, *p* < 0.05, η^2^_P_ = 0.851). Formulated Sentences also present challenges for G3 (M = 4.54, SD = 2.943) compared to G1 (M = 16.06, SD = 3.909) and G2 (M = 23.54, SD = 3.945), with a significant difference (F = 347.85, *p* < 0.05, η^2^_P_ = 0.826), indicating difficulties in constructing coherent sentences. Likewise, Recalling Sentences reveals lower performance in G3 (M = 3.72, SD = 2.872) compared to G1 (M = 15.72, SD = 4.010) and G2 (M = 24.78, SD = 4.674), with a significant difference (F = 362.51, *p* < 0.05, η^2^_P_ = 0.831), suggesting challenges in recalling and reproducing sentences accurately. Furthermore, Understanding Spoken Paragraphs shows G3 (M = 4.44, SD = 2.786) with inferior performance relative to G1 (M = 16.26, SD = 3.942) and G2 (M = 23.70, SD = 3.955), with a significant difference (F = 363.31, *p* < 0.05, η^2^_P_ = 0.832), indicating difficulties in comprehending longer verbal passages. Word Definitions also present challenges for G3 (M = 4.23, SD = 2.954) compared to G1 (M = 15.94, SD = 3.582) and G2 (M = 25.45, SD = 4.476), with a significant difference (F = 395.64, *p* < 0.05, η^2^_P_ = 0.864), suggesting limitations in defining and understanding word meanings. Additionally, Sentence Assembly reveals G3 (M = 4.73, SD = 2.621) with lower performance compared to G1 (M = 16.02, SD = 3.877) and G2 (M = 24.49, SD = 4.258), with a significant difference (F = 357.45, *p* < 0.05, η^2^_P_ = 0.851), indicating challenges in assembling sentences logically. Lastly, the Pragmatics Profile demonstrates G3 (M = 4.44, SD = 2.830) with inferior performance relative to G1 (M = 16.90, SD = 4.414) and G2 (M = 23.80, SD = 3.725), with a significant difference (F = 349.14, *p* < 0.05, η^2^_P_ = 0.826), suggesting difficulties in using language appropriately in social contexts. These findings underscore the heightened difficulties in various aspects of language observed within the G3 group in contrast to the individual groups G1 and G2.

## 5. Discussion

The objective of this research was to examine and contrast potential variations in both language and executive functioning across three distinct groups: individuals diagnosed solely with ASD, those diagnosed solely with epilepsy, and those diagnosed with the comorbidity of ASD and epilepsy during childhood. The primary hypothesis posited that individuals diagnosed with epilepsy would demonstrate superior scores on assessments of executive functioning in comparison to those with ASD or the comorbid condition of ASD and epilepsy. The secondary hypothesis suggested that individuals with epilepsy would display enhanced performance and higher scores across language dimensions relative to individuals with ASD or the comorbid condition of ASD and epilepsy.

Below, discussions of the results regarding executive functioning and language skills obtained by the three groups of participants in the present study are carried out.

### 5.1. Discussion of Results Regarding Participants’ Executive Functioning

In terms of executive functioning abilities, the findings indicated that the epilepsy group outperformed both the ASD group and the group with comorbid ASD and epilepsy. Moreover, the epilepsy group demonstrated superior skills in executive functioning, including planning, organization, working memory, inhibition, and emotional control, among others. These competencies enable them to carry out daily tasks more effectively compared to children with ASD and epilepsy. Consequently, individuals with epilepsy but without ASD generally exhibit better performance in executive functioning processes, leading to an enhanced development of daily life activities and autonomy in various settings such as home or school. It is suggested that the presence of ASD may impede the proper development of certain executive functioning processes, like cognitive flexibility, planning, organization, inhibition, or emotional control, which significantly affects the execution of basic daily activities across different contexts. However, when ASD and epilepsy coexist in children, challenges in executive functioning become more pronounced due to impairments in specific brain regions, such as the prefrontal and temporal lobes.

The current study identified a notable pattern indicating that individuals with ASD and epilepsy exhibit compromised executive functions in comparison to other participants. This suggests that ASD has a detrimental effect on these executive skills, consequently impeding autonomy, learning, and integration across various daily life contexts. This observation is corroborated by studies such as Pisón et al. [25], which demonstrate that children diagnosed with both ASD and epilepsy display more pronounced cognitive impairment and poorer executive functioning when compared to those with ASD alone.

Regarding tasks such as information processing, emotional control, attention, and working memory, the study by Pisón et al. [25] also indicates that this population usually experiences severe difficulties in all these areas. These results are similar to those of Barboza Ubarnes et al. [41], where it is demonstrated that children with ASD and epilepsy often exhibit a symptomatic pattern highlighting difficulties in executive functioning, specifically in working memory, verbal information processing, and emotional control. This is attributed to focal damage in brain areas related to executive functions, such as the frontal lobe. However, these studies do not conduct a comparative analysis between language and executive functioning competencies but offer a general view of the alterations presented by these profiles without establishing a relationship between the two competencies. Therefore, executive functioning skills might be more pronounced in children with ASD who also have epilepsy, potentially reducing their abilities in cognitive flexibility, planning, organization, working memory, inhibition, or emotional control [60,61,62].

### 5.2. Discussion of Results Regarding Participants’ Language Skills

Regarding language skills, the results obtained in the present study confirm the previously established research hypothesis. This is because participants who had epilepsy as the primary diagnosis demonstrated better performance in communicative and linguistic abilities, showing a linguistic profile characterized by better performance in tasks related to Sentence Comprehension, Linguistic Concepts, Word Structure, Semantic Relationships, Following Directions, Formulated Sentences, Recalling Sentences, Understanding Spoken Paragraphs, Word Definitions, Sentence Assembly, and Pragmatics Profile, compared to children diagnosed with ASD or ASD with epilepsy. Additionally, the results indicate that the presence of ASD alongside epilepsy leads to limitations in language, communication, and speech performance. The pediatric population with ASD exhibits atypical development in terms of language aspects and dimensions, where alterations in speech sound articulation and its phonological and phonetic processing, acquisition of functional vocabulary and its integration into their lexical-semantic store, grammatical coherence, and cohesion with morphosyntactic structures adjusted to their chronological age, and the interaction and use of language through gestures, communicative intention, or narrative related to pragmatics can be observed [60]. It seems that epilepsy located in the major areas of expressive and receptive language causes a delay in acquiring linguistic competencies in this pediatric population with ASD during the early stages of maturation. This is manifested in the lack of proper theory of mind, difficulties in relating to peers, as well as expressing desires or needs [61,62,63].

Similarly, in the present study, alterations are observed in grammatical processes, sentence repetition, elaboration of morphosyntactic structures, and difficulties in lexical access and vocabulary. All of this leads participants with ASD and epilepsy to show limited performance in linguistic processes, both in their expressive and receptive dimensions, thus limiting their interaction with the environment and exacerbating linguistic characteristics already observed in epilepsy as the primary diagnosis. These results align with current research, such as that conducted by del Busto [42] and Campos-Castelló and Campos-Soler [36], which indicate that the pediatric population with ASD and epilepsy often presents alterations in lexical access processes, as well as in the pragmatic dimension of language.

Communication difficulties in children with epilepsy have been noted in studies such as Zambarbieri [43], which highlight the vulnerability of areas such as the temporal and frontal lobes to epileptic foci. Consequently, deficits in semantic memory and language, including reduced verbal fluency and anomia are frequently reported. When epilepsy coexists with ASD, these linguistic challenges appear to be more pronounced [40]. This suggests that individuals with ASD and epilepsy face heightened limitations in communication and language, necessitating specialized care, as emphasized in this study. Support for this notion is evident in research by Dioses-Chocano et al. [44], Juillerat et al. [45], Norrelgen et al. [46], and Rotta [47], which examine language skills in pediatric populations with ASD or ASD with epilepsy. These studies consistently indicate greater language impairments in children with ASD and epilepsy compared to those without this comorbidity. Furthermore, it is suggested that this population experiences difficulties in acquiring first words, mastering grammatical and syntactic language forms, and engaging in functional conversation with their surroundings. Consequently, the present study underscores that children meeting this profile often exhibit delays in sentence production prior to entering primary school. Therefore, compelling evidence from this study suggests that children diagnosed with both ASD and epilepsy tend to experience more pronounced delays in acquiring oral communication skills compared to their peers without epilepsy [64].

### 5.3. Discussion of the Results from the Perspective of Educational Neuroscience

Adverse outcomes in children’s cognitive and linguistic development can be attributed to the ongoing interaction of toxic stressors impacting the brain both during pregnancy and the child’s first 1000 days of life. These stressors may include environmental exposures such as air pollution, household or community toxin exposure, prenatal stress, and inadequate nutrition, among others. Exposure to these stressors during critical periods of brain development can have significant and lasting consequences on brain structure and function, which in turn, can negatively affect the child’s cognitive and linguistic development. In this context, it is emphasized how decisions made by the school team in collaboration with families can be crucial in designing individualized educational plans that accommodate each child’s strengths and weaknesses. These plans include specific interventions to address areas of development affected by identified stressors, such as early intervention programs, speech and language therapy, and emotional and psychological support. However, to further enrich this educational strategy, it would be beneficial to establish close collaboration with healthcare professionals. These professionals offer valuable diagnostic insights based on the origins of language and cognitive-related educational challenges, considering both endogenous and exogenous stressors, such as interactions between genes and the environment. For example, a pediatrician may identify and address underlying health issues contributing to the child’s developmental challenges, while a pediatric neurologist can assess and treat specific neurological disorders affecting cognitive and linguistic development. Additionally, an interdisciplinary perspective spanning neuroscience, psychology, education, and medicine provides a more comprehensive understanding of factors influencing children’s cognitive and linguistic development and allows for a more holistic approach to intervention and treatment. This interdisciplinary collaboration would include regular meetings among educators, physicians, and other healthcare professionals to review the child’s progress, share relevant information, and coordinate interventions to ensure comprehensive, child-centered care.

Furthermore, it would be valuable to examine how these recent research findings relate to the findings of the present study and how they can inform our interpretations and recommendations. For example, considering the healthcare disparities highlighted by Scher [65], we could reflect on how these disparities may affect access to early intervention services and specialized healthcare for children at risk of cognitive and linguistic challenges. This could include a lack of access to specialized assessments and treatments, which could, in turn, delay identification and early intervention in children with specific needs, directly influencing their long-term cognitive and linguistic development. Similarly, by exploring the role of the neuronal exposome in childhood development according to Scher [66], we could reflect on how environmental exposures and stressors may interact with biological processes to influence brain development and ultimately cognitive and linguistic skills in children with ASD and epilepsy. This could involve considering how environmental pollution, prenatal stress, and other environmental factors may negatively impact brain development and cognitive function in this population, which could have significant consequences for their ability to acquire and develop language and other cognitive skills. These reflections may lead to recommendations for policies and practices that address healthcare inequities and promote healthy and safe environments for child development. For example, policies could be suggested to increase equitable access to healthcare and education services, as well as community interventions aimed at reducing exposure to environmental stressors and promoting healthy parenting environments. Ultimately, a deeper understanding of these issues can lead to more effective strategies to support optimal cognitive and linguistic development in children and address disparities in child health and well-being.

## 6. Conclusions

In summary, individuals with ASD and epilepsy demonstrate heightened deficits in executive functions such as inhibition, flexibility, emotional control, working memory, planning, organization, and monitoring, along with challenges in language areas including phonology, semantics, morphosyntax, and pragmatics, when compared to those with ASD alone. It is evident that children affected by these conditions encounter obstacles in both executive functioning and expression as well as receptive language abilities. These challenges stem from disruptions in cognitive-linguistic capabilities, impacting overall functioning.

However, this study faces limitations related to the choice of instruments, which are objective and not reliant on self-reports. The BRIEF-2 is employed to assess executive functions, utilizing a questionnaire completed by family members based on their observations and experiences within the child’s developmental environment. While offering considerable ecological validity, the BRIEF-2 could benefit from the inclusion of additional tests administered directly to participants by examiners. However, the ENFEN test was indeed administered by a neuropsychologist, allowing for the control of potential bias found in the self-administration of the BRIEF-2 questionnaire by the family members and teachers of the participants comprising the sample group in this study. Moreover, it is important to acknowledge the variability in clinical and symptomatic profiles among individuals diagnosed with epilepsy, ASD, or both. A thorough analysis of alterations based on the severity of ASD within these populations is warranted. Additionally, an evaluation within an academic setting is proposed to enable comparisons between assessment scores, parental reports, and feedback from teachers.

Despite these limitations, this work paves the way for future research to address these deficiencies. Furthermore, other subtypes of ASD should be evaluated for a more profound comparison of this disorder. Different types of epilepsy should also be compared, as well as conducting neuroimaging studies to allow comparisons based on the location of the epileptic focus in the pediatric population with this condition. Along the same lines, a research avenue is proposed to describe emotional alterations or the quality of life of the family nucleus. The information collected in the parental references of the selected populations in this study would help determine the linguistic and cognitive maturation of these three groups of individuals. Additionally, it is essential to carry out additional assessments of other factors, such as motor skills and sensory profiles, which are crucial in the ASD population. Lastly, future research could incorporate the examination of social skills and their development across various forms of ASD, comparing them with other comorbid disorders.

In summary, the rising prevalence of coexisting ASD and epilepsy highlights the importance of identifying and monitoring various factors and characteristics for early intervention in the cognitive-linguistic impairments associated with this dual condition. The concurrent presence of ASD and epilepsy may hinder the optimal development of affected children, impacting their executive and language skills. As a result, these limitations could impede the smooth execution of daily activities, reducing the efficiency and autonomy of individuals with ASD with epilepsy.

## Figures and Tables

**Table 1:** Characteristics of the study participants.

	Epilepsy Group (*n* = 50)	ASD Group (*n* = 50)		ASD with Epilepsy Group (*n* = 50)	Chi-Square (*p*)
Gender					1.025 (0.599)
Men (%)	28 (56%)	25 (50%)		30 (60%)	
Female (%)	22 (44%)	25 (50%)		20 (40%)	
Treatment Duration					5.529 (0.237)
2 years (%)	16 (32%)	16 (32%)	18 (36%)		
3 years (%)	16 (32%)	17 (34%)	17 (34%)		
4 years (%)	18 (36%)	17 (34%)	15 (30%)		
Grade of Disability				5.756 (0.218)	
Less than 33%	17 (34%)	17 (34%)	16 (32%)		
Between 33% and 66%		15 (30%)		16 (32%)	17 (34%)
More than 66%	18 (36%)	17 (34%)	17 (34%)		
Educational supports at school				6.492 (0.165)	
Speech and Language Teacher (%)	15 (30%)	15 (30%)	20 (40%)		
Speech and Language Teacher + Special Education Teacher (%)	17 (34%)	17 (34%)	16 (32%)		
Speech and Language Teacher + Special Education Teacher + Educator (%)	18 (36%)	18 (36%)	14 (28%)		
Gestation Weeks				2.114 (0.715)	
30–35 weeks (%)	18 (36%)	17 (34%)	15 (30%)		
35–40 weeks (%)	16 (32%)	16 (32%)	18 (36%)		
>40 weeks (%)	16 (32%)	17 (34%)	17 (34%)		
Apgar Score				0.584 (0.965)	
At risk (%)	16 (32%)	17 (34%)	14 (28%)		
Intermediate (%)	17 (34%)	16 (32%)	17 (34%)		
Normal (%)	17 (34%)	17 (34%)	19 (38%)		
Medication				7.447 (0.281)	
No presence (%)	16 (32%)	17 (34%)	17 (34%)		
Valproate (%)	17 (34%)	16 (32%)	17 (34%)		
Risperidone (%)	17 (34%)	18 (36%)	15 (30%)		
Clonazepam (%)	17 (34%)	16 (32%)	18 (36%)		
Intellectual disability				34.584 (0.001)	
Non-presence	50 (41%)	45 (37%)	28 (22%)		
Mild intellectual disability	0 (0%)	5 (18%)	22 (82%)		
Total mean IQ (Mean)	91.4	87.2	78.1		

**Table 2:** Comparative analysis among groups: ASD (G1), Epilepsy (G2), and ASD and epilepsy (G3) in executive functioning measures assessed in the BRIEF-2 test.

	ASD (*n* = 50)		Epilepsy (*n* = 50)		ASD and Epilepsy (*n* = 50)		F_(2,147)_	η^2^_P_	Difference between Groups
	M	SD	M	SD	M	SD			
Inhibition	14.86	3.60	23.58	3.74	4.50	2.97	382.16 *	0.839	G3 < G1 < G2
Flexibility	13.42	3.74	23.92	3.70	4.72	2.90	382.53 *	0.839	G3 < G1 < G2
Emocional control	13.20	3.06	24.16	3.54	4.50	2.95	475.18 *	0.866	G3 < G1 < G2
Work memory	14.00	3.85	23.98	4.06	4.58	2.95	351.65 *	0.827	G3 < G1 < G2
Planning	14.12	3.79	23.60	3.54	4.10	2.83	406.85 *	0.847	G3 < G1 < G2
Organization of materials	13.80	3.79	23.36	3.83	4.48	3.07	347.30 *	0.825	G3 < G1 < G2
Monitoring	13.90	3.53	24.66	3.52	4.52	2.94	453.46 *	0.861	G3 < G1 < G2

* *p* < 0.05.

**Table 3:** Comparative analysis among groups: ASD (G1), epilepsy (G2), and ASD and epilepsy (G3) in executive functioning measures assessed in the ENFEN test.

	ASD (*n* = 50)		Epilepsy (*n* = 50)		ASD and Epilepsy (*n* = 50)		F_(2,147)_	η^2^_P_	Difference between Groups
	M	SD	M	SD	M	SD			
Verbal fluency	16.82	3.37	23.84	5.25	9.40	3.37	155.27 *	0.679	G3 < G1 < G2
Trail construction	18.26	3.48	23.74	5.11	8.26	3.16	191.16 *	0.722	G3 < G1 < G2
Ring construction	17.48	3.88	24.46	5.05	8.06	3.01	204.40 *	0.736	G3 < G1 < G2
Interference resistance	18.22	3.77	24.12	5.39	8.20	2.99	185.84 *	0.717	G3 < G1 < G2

* *p* < 0.05.

**Table 4:** Comparative analysis among ASD (G1), epilepsy (G2), and ASD with epilepsy (G3) groups based on language measures derived from the CELF-5 assessment.

	ASD (*n* = 50)		Epilepsy (*n* = 50)		ASD and Epilepsy (*n* = 50)		F_(2,147)_	η^2^_P_	Difference between Groups
	M	SD	M	SD	M	SD			
Sentence Comprehension	14.92	3.680	25.06	4.133	4.44	2.908	408.05 *	0.847	G3 < G1 < G2
Linguistic Concepts	15.84	3.776	24.40	3.860	4.40	3.117	388.55 *	0.841	G3 < G1 < G2
Word Structure	16.36	4.355	23.50	3.477	4.12	3.055	356.72 *	0.829	G3 < G1 < G2
Semantic Relationships	16.58	3.866	24.18	4.183	4.72	2.865	354.96 *	0.828	G3 < G1 < G2
Following Directions	15.60	3.725	25.36	4.070	4.48	2.936	419.14 *	0.851	G3 < G1 < G2
Formulated Sentences	16.06	3.909	23.54	3.945	4.54	2.943	347.85 *	0.826	G3 < G1 < G2
Recalling Sentences	15.72	4.010	24.78	4.674	3.72	2.872	362.51 *	0.831	G3 < G1 < G2
Understanding Spoken Paragraphs	16.26	3.942	23.70	3.955	4.44	2.786	363.31 *	0.832	G3 < G1 < G2
Word Definitions	15.94	3.582	25.45	4.476	4.23	2.954	395.64 *	0.864	G3 < G1 < G2
Sentence Assembly	16.02	3.877	24.49	4.258	4.73	2.621	357.45 *	0.851	G3 < G1 < G2
Pragmatics Profile	16.90	4.414	23.80	3.725	4.44	2.830	349.14 *	0.826	G3 < G1 < G2

* *p* < 0.05.

## Data Availability

The data presented in this study are available on request from the corresponding author. The data are not publicly available due to to specific ethical and privacy considerations.
